# Event‐related potentials in response to feedback following risk‐taking in the hot version of the Columbia Card Task

**DOI:** 10.1111/psyp.13390

**Published:** 2019-05-08

**Authors:** Kristel de Groot, Jan W. van Strien

**Affiliations:** ^1^ Erasmus University Rotterdam Institute for Behaviour and Biology (EURIBEB) Erasmus University Rotterdam Rotterdam The Netherlands; ^2^ Institute of Psychology, Erasmus School of Social and Behavioural Sciences Erasmus University Rotterdam Rotterdam The Netherlands; ^3^ Department of Applied Economics, Erasmus School of Economics Erasmus University Rotterdam Rotterdam The Netherlands

**Keywords:** Columbia Card Task, electroencephalography, FRN, P300, risk

## Abstract

Given the importance of risk‐taking in individuals’ personal and professional life, several behavioral tasks for measuring the construct have been developed. Recently, a new task was introduced, the Columbia Card Task (CCT). This task measures participants’ risk levels and establishes how sensitive participants are to gains, losses, and probabilities when taking risk. So far, the CCT has been examined in behavioral studies and in combination with several (neuro)biological techniques. However, no electroencephalography (EEG) research has been done on the task. The present study fills this gap and helps to validate this relatively new experimental task. To this end, *n* = 126 students were asked to complete self‐reports (reward responsiveness, impulsiveness, and sensation‐seeking) and to perform the CCT (and other risk tasks) in an EEG setup. The results show that feedback appraisal after risky decision‐making in the CCT was accompanied by a feedback‐related negativity (FRN) and a P300, which were stronger in response to negative than positive feedback. Correlations between the FRN and P300 difference wave on the one hand and risk‐related self‐reports and behavior on the other were nonsignificant and small, but were mostly in the expected direction. This pattern did not change after excluding participants with psychiatric/neurological disorders and outliers. Excluding participants with reversed (positive > negative) difference waves strengthened FRN correlations. The impact such individuals can have on the data should be taken into account in future studies. Regarding the CCT in particular, future studies should also address its oddball structure and its masking of true values (censoring).

## Abstract 2

This article aids in validating a new risk task, the Columbia Card Task (CCT), by examining its event‐related potentials (ERPs). The CCT has not been used in combination with electroencephalography (EEG) before, nor with any other recording technique with a high temporal resolution. Two ERPs were observed during feedback appraisal: the feedback‐related negativity (FRN) and the P300. By relating these ERPs to self‐reports and behavior, the article contributes to the literature on feedback‐related ERPs. It also contributes to CCT literature by identifying key concerns regarding the behavioral and electrophysiological application of the CCT and by offering possible solutions.

## 1 INTRODUCTION

Since deciding whether or not to take a risk can have large consequences for personal and professional ventures, risk‐taking propensity is examined in multiple scientific fields, such as neuroscience, psychology, criminology, economics, and management. For this purpose, several (computerized) behavioral tasks for measuring the construct have been developed. Well‐known tasks include the Iowa Gambling Task (IGT; Bechara, Damasio, Damasio, & Anderson, 1994), the Balloon Analogue Risk Task (BART; Lejuez et al., 2002), the Cambridge Gambling Task (CGT; Rogers et al., 1999), and the Game of Dice Task (GDT; Brand et al., 2005). Recently, Figner, Mackinlay, Wilkening, and Weber (2009) introduced a new computer task to measure risk‐taking propensity: the Columbia Card Task (CCT).

In the CCT, participants turn virtual cards from a 32‐card array. Most of these cards are win cards, which earn participants points. However, a small number of cards are loss cards, which make them lose points. In every trial, participants are given three information parameters to help them decide how many cards to turn: (1) the number of points they gain when turning a win card, (2) the number of points they lose when encountering a loss card, and (3) the number of loss cards present in the trial. The way in which cards are turned differs across task versions. In the so‐called “hot” CCT, participants turn the cards one by one (thereby accumulating points) until they voluntarily stop and cash the points or until they turn a loss card, at which point the specified loss amount is subtracted from the points earned (and the remainder is cashed). In the “cold” CCT, however, participants indicate at the start of every trial how many cards they want to turn, after which the computer determines the trial's outcome, unseen by the participant. The key difference between the versions is whether or not participants receive feedback following their choices. In the cold CCT, participants get to see only the final result of the game, which elicits deliberative decision‐making. In the hot CCT, they receive feedback after every card turn, eliciting affective decision‐making. Later work by Huang, Wood, Berger, and Hanoch (2013) introduced a third, “in‐between” version: the “warm” CCT. Here, participants select the cards they would like to turn at the start of a trial and then press a button that prompts the selected cards to turn, thereby providing delayed feedback.

Regardless of which version, the CCT is characterized by two advantages. First, since the task gives participants information on the probability of losing (i.e., the number of loss cards present in a trial), it is an apparent risk task that leaves no room for conceptual ambiguity (de Groot & Thurik, 2018). This is different from, for example, the BART (where people are unaware of probabilities and therefore decide under uncertainty) and the IGT (where people learn the probabilities while progressing through the task and thus gradually shift from making decisions under uncertainty to making decisions under risk). The second advantage of the CCT is its use of a so‐called full factorial design, which independently varies the three information parameters given to participants (the number of points awarded for turning win cards, the number of points subtracted when turning a loss card, and the number of loss cards present) across trials so that all possible combinations are presented a given number of times. This design prevents risk and expected value from being confounded. In the IGT, BART, CGT, and GDT, (presumed) riskier options have a lower expected value than less risky options (Figner et al., 2009; Schonberg, Fox, & Poldrack, 2011). This causes a decomposition problem, since it is unclear to what extent someone's observed level of risk‐taking is driven by risk attitude (information on probabilities), sensitivity to reward (information on gains), or sensitivity to loss (information on losses). Its full factorial design enables the CCT to establish the extent to which these factors separately affect a participant's level of risk‐taking.

The CCT's advantages have so far been used in several behavioral studies and in combination with various (neuro)biological techniques. For example, increased risk‐taking in the cold CCT has been associated with higher impulsivity (Penolazzi, Gremigni, & Russo, 2012) and more errors in an executive function task (Buelow, 2015). Increased risk‐taking in the hot CCT has been related to high grandiosity (Brunell & Buelow, 2017) and reward responsiveness (Penolazzi et al., 2012). Using both the cold and hot CCT, several studies have found dissociations. For example, adolescents took more risk than adults in the hot but not in the cold CCT (Figner et al., 2009). A similar pattern was observed for patients with ventromedial prefrontal cortex (VMPFC) lesions compared to healthy controls (Spaniol, di Muro, & Ciaramelli, 2018). Biological dissociations have also been reported: electrodermal activity (EDA) only increased from baseline to decision phase in the hot and not the cold version of the task (Figner et al., 2009). Extending this finding, Holper and Murphy (2014) showed an opposite pattern for EDA and brain activity as measured with functional near‐infrared spectroscopy (fNIRS): whereas skin conductance was larger in the hot than in the cold CCT, prefrontal total hemoglobin concentration (tHb) changes were larger in the cold version of the task. Another dissociation was reported in a study on hemispheric asymmetry using transcranial direct current stimulation (tDCS), showing that anodal left/cathodal right but not anodal right/cathodal left stimulation over the dorsolateral prefrontal cortex (DLPFC) decreased risk‐taking in the cold CCT, which fits with the hypothesized involvement of the left DLPFC in deliberative information processing (Pripfl, Neumann, Köhler, & Lamm, 2013).

In addition to these findings on absolute risk levels, several studies have examined how individuals use the information (on gains, losses, and probabilities) that is provided to them in every trial. Distinctive patterns of information use have, for example, been observed in adolescents (Figner et al., 2009) and older adults (Huang, Wood, Berger, & Hanoch, 2015): both were shown to take less information into account than young and middle‐aged adults when making decisions in the hot and warm CCT, respectively. Aberrant sensitivity to information has also been observed in several patient groups: compared to healthy controls, crack cocaine users (Kluwe‐Schiavon, Viola, Sanvicente‐Vieira, Pezzi, & Grassi‐Oliveira, 2016), heroin‐dependent persons (Saleme et al., 2018), and individuals with lesions in the VMPFC (Spaniol et al., 2018) paid less attention to probabilities. Decreased information sensitivity has also been reported in healthy individuals. For example, the positive association between the use of habitual cognitive reappraisal and risk‐taking in the cold CCT as reported by Panno, Lauriola, and Figner (2013) was accompanied by reduced sensitivity to loss and probability information, suggesting that reappraisal operates via decreasing the attention to the negative aspects of a choice. Likewise, Penolazzi et al. (2012) showed that the association between reward responsiveness and risk‐taking in the hot CCT interacted with information on gains and losses in such a way that individuals who scored high on reward responsiveness were sensitive to high gains while neglecting concomitant high loss. These studies, among others, clearly illustrate the benefits of the CCT's full factorial design by examining not only risk behavior itself but also the motives that drive it.

Whereas many of the older decision tasks (such as the BART and the IGT) have been explored with electroencephalography (EEG), no EEG research has yet been done on the CCT. Previous EEG research on the BART (e.g., Kardos et al., 2016; Kessler, Hewig, Weichold, Silbereisen, & Miltner, 2017; Takács et al., 2015) and the IGT (e.g., Mapelli, Di Rosa, Cavalletti, Schiff, & Tamburin, 2014; Oberg, Christie, & Tata, 2011; Tamburin et al., 2014) primarily focused on the feedback phase of the tasks in which the rapid appraisal of the decision outcomes is usually captured by two event‐related potentials (ERPs): the feedback‐related negativity (FRN) and the feedback‐related positivity 300 (P300). Since previous (neuro)biological research on the CCT employed measures with lower temporal resolution (EDA [Figner et al., 2009; Holper & Murphy, 2014], fMRI [van Duijvenvoorde et al., 2015], and fNIRS [Holper & Murphy, 2014]) or focused on stimulating rather than recording brain activity (tDCS; Pripfl et al., 2013), examining the ERPs for the CCT could aid in validating this relatively new experimental task.

The first ERP of interest, the FRN, is a negative deflection peaking at frontocentral sites, and reaches its maximum 200–300 ms after feedback presentation (Holroyd & Coles, 2002; Miltner, Braun, & Coles, 1997). Generation of the potential is closely linked to the mesolimbic dopaminergic system (Nieuwenhuis, Holroyd, Mol, & Coles, 2004; Walsh & Anderson, 2012). When an outcome is worse than expected, mesencephalic dopaminergic firing decreases (Holroyd & Coles, 2002). These transient dopaminergic dips signal disinhibition of apical dendrites in the anterior cingulate cortex (ACC), which uses the signal to determine the most suitable behavior for the situation at hand. The FRN reflects an early and rapid bad versus good evaluation of feedback. Accordingly, it is influenced by only the valence and not by the magnitude of rewards, showing stronger amplitudes following negative than following positive feedback (Hajcak, Moser, Holroyd, & Simons, 2006; Miltner et al., 1997; Yeung & Sanfey, 2004). With regard to risk‐taking, stronger amplitudes have been related to increased risk aversion (Schuermann, Endrass, & Kathmann, 2012); blunted absolute and relative (difference) waves have been observed in individuals who typically take more risk, such as people dealing with borderline personality disorder (Endrass, Schuermann, Roepke, Kessler‐Scheil, & Kathmann, 2016), family alcohol problems (Fein & Chang, 2008), or problematic internet use (Yau, Potenza, Mayes, & Crowley, 2015). These findings suggest a relationship between increased risk‐taking and underdeveloped internal models and warning signals. In line with this, larger FRN difference waves have been associated with higher executive function (Kóbor et al., 2015) and a preference for low‐risk decisions (Endrass et al., 2016).

The FRN is typically followed by the second ERP of interest: the P300, a positive, parietally distributed deflection that peaks approximately 300–500 ms after feedback presentation (Kopp & Wolff, 2000; Sutton, Braren, Zubin, & John, 1965). This potential is linked to the noradrenergic system and hence to locus coeruleus activity (Nieuwenhuis, Aston‐Jones, & Cohen, 2005; Polich, 2007). Candidate regions for its neural basis are the cingulate cortex and adjacent areas involved in the circuit between frontal and parietal regions (Linden, 2005; Nieuwenhuis et al., 2005). Contrary to the FRN, the P300 reflects elaborate appraisal of feedback, varies with the motivational significance of this feedback (Kleih, Nijboer, Halder, & Kübler, 2010; Nieuwenhuis et al., 2005), and is sensitive to top‐down attentional control (Gray, Ambady, Lowenthal, & Deldin, 2004; Nieuwenhuis et al., 2005; Polich, 2007). The literature is inconsistent as to whether the P300 is sensitive to valence. Some studies indicate no effect (Yeung & Sanfey, 2004), whereas others show a stronger P300 following positive feedback (Wu & Zhou, 2009; Zhou, Yu, & Zhou, 2010) or negative feedback (Crowley et al., 2009; Endrass et al., 2016; Euser, Evans, Greaves‐Lord, Huizink, & Franken, 2013; Euser, Greaves‐Lord, et al., 2013; Fein & Chang, 2008; Kóbor et al., 2015; Schuermann et al., 2012). With regard to risk‐taking, absolute amplitudes are larger for high‐risk than for low‐risk decisions (Endrass et al., 2016; Schuermann et al., 2012). These higher amplitudes, especially in response to negative feedback, are related to greater risk avoidance. Reduced (absolute and difference) waves are observed in risk‐prone people, such as people who are alcohol‐intoxicated (Euser, van Meel, Snelleman, & Franken, 2011) and individuals who have a parental history of substance abuse (Euser, Greaves‐Lord, et al., 2013), show features of problematic internet use (Yau et al., 2015), or are diagnosed with borderline personality disorder (Endrass et al., 2016). Blunted absolute P300s may reflect a diminished ability to engage in feedback appraisal, outcome prediction, and cognitive control. Reduced difference scores can be the result of a heightened response to gains and/or a weaker response to losses.

The present study aids in validating the CCT by examining ERPs in response to feedback in the hot version of the task. The hot version is particularly suitable for this purpose, since the cold CCT does not provide feedback, and since the later‐developed warm CCT (which does provide feedback) is still in a pioneering phase. First, we examine the FRN and P300, two ERPs that are commonly observed during feedback appraisal in other behavioral risk tasks, such as the BART and the IGT (Kessler et al., 2017; Oberg et al., 2011). Based on this literature, we expected that feedback appraisal in the hot CCT would also by accompanied by an FRN and a P300, and that both potentials would be more potent following negative than following positive feedback. Second, we examine the correlations between the observed CCT ERPs and risk‐related self‐reports and behavior. Here, the absolute ERPs are transformed into difference waves by subtracting the positive feedback‐locked waveform from the negative one (see, e.g., Fein & Chang, 2008; Kóbor et al., 2015). The advantage of doing so is twofold: it eliminates exogenous components—that is, elements that are elicited in response to all stimuli and hence across all conditions (Miltner et al., 1997)—and it corrects for individual differences in general wave amplitude, given that absolute waves may reflect a general tendency for small or large amplitudes, rather than the underlying construct (which is especially problematic for correlations). Correlations are calculated between the ERP difference waves and the following variables: behavioral measures derived from the hot CCT itself (average number of card turns; number of loss card encounters; sensitivity to gain, loss, and probability); risk‐taking on the cold CCT and the BART (which has been shown to correlate with risk‐taking on the hot CCT: Buelow & Blaine, 2015; Saleme et al., 2018); gender (which has been shown to correlate with several tasks and types of risk‐taking and which has been related to the FRN and P300: Byrnes, Miller, & Schafer, 1999; Ding et al., 2017; Hirayasu, Samura, Ohta, & Ogura, 2000); and three self‐report constructs that have been related to either hot CCT behavior itself or to behavior and electrophysiology in other tasks probing affective decision‐making: reward responsiveness (Penolazzi et al., 2012) and impulsivity and sensation‐seeking (Euser et al., 2011; Lejuez et al., 2002). Based on this literature, we hypothesized that reduced FRN and P300 difference scores (i.e., smaller differences between responses to negative and positive feedback) are associated with the following variables: being male; higher risk‐taking in the BART, the cold CCT, and the hot CCT; a higher number of loss cards encountered; higher reward responsiveness, impulsivity, and sensation‐seeking; increased sensitivity to gains; and lower sensitivity to losses and probabilities.

## 2 METHOD

### 2.1 Participants

The sample consisted of *n* = 126 students (52.38% female) recruited from two universities, with a mean age of *M* = 21.01 (*SD* = 2.62), range 17–31 years. Most participants were studying social sciences (38.89%), economics (26.19%), or management (19.84%), although all main fields of study (including law, mathematics, and medicine) were represented. In exchange for participation, students received either course credit or a standard fee of €25. They were informed that they could earn extra money (up to €7.50) based on their task performance. All participants provided written informed consent. The study was approved by the institutional review board, and all procedures performed were in accordance with the 1964 Helsinki declaration and its later amendments.

### 2.2 Procedure

The measures were part of a larger study on decision‐making under uncertain and risky conditions. Participants signed up online based on a brief description of the study design, after which they received an email with more elaborate information and the request to not drink alcohol, coffee, or energy drinks on the day of the appointment to prevent these substances from impacting the measurements. This email also contained a link to a web‐based survey including the self‐report measures, which participants were required to complete before their appointment at the laboratory. During the appointment itself, the procedure was explained to the participant, and the participant was asked to provide written informed consent. Then the participant was seated in a light‐ and sound‐attenuated EEG room, was wired to the electrodes, and was presented with the BART and both CCTs. The order in which the tasks were presented was counterbalanced across participants. After finishing the tasks, the participant was debriefed. The full session lasted approximately 1.5 hr.

### 2.3 Self‐report measures

#### 2.3.1 Reward responsiveness

Reward responsiveness (RR) was measured using the RR subscale of the Behavioral Approach System (BAS) questionnaire (Carver & White, 1994). This subscale consists of five items (4, 7, 14, 18, and 23) and is answered on a 4‐point scale with labels “completely disagree,” “disagree,” “agree,” and “completely agree.” The RR score ranges from 5 to 20, with higher scores indicating higher trait reward responsiveness. In the present study, scores ranged from 9 to 20. Cronbach's alpha was α = 0.68.

#### 2.3.2 Impulsiveness

Impulsiveness was measured using the Barratt Impulsiveness Scale 11 (BIS‐11; Patton, Stanford, & Barratt, 1995), which consists of 30 items that are answered on a 4‐point scale with labels “rarely/never,” “occasionally,” “often,” and “almost always/always.” The BIS‐11 score ranges from 30 to 120, with higher scores being indicative of higher trait impulsiveness. Scores in the present study ranged from 43 to 87. Cronbach's alpha was α = 0.76.

#### 2.3.3 Sensation‐seeking

Sensation‐seeking was measured using the Brief Sensation Seeking Scale (BSSS; Hoyle, Stephenson, Palmgreen, Lorch, & Donohew, 2002), which consists of eight items that are answered on a 5‐point scale with labels “strongly disagree,” “disagree,” “neither disagree nor agree,” “agree,” and “strongly agree.” The BSSS scale ranges from 8 to 40, with higher scores indicating higher trait sensation‐seeking. Scores in the present study ranged from 11 to 37. Cronbach's alpha was α = 0.77.

### 2.4 Behavioral tasks

#### 2.4.1 Automatic Balloon Analogue Risk Task (BART)

**Figure 1:**
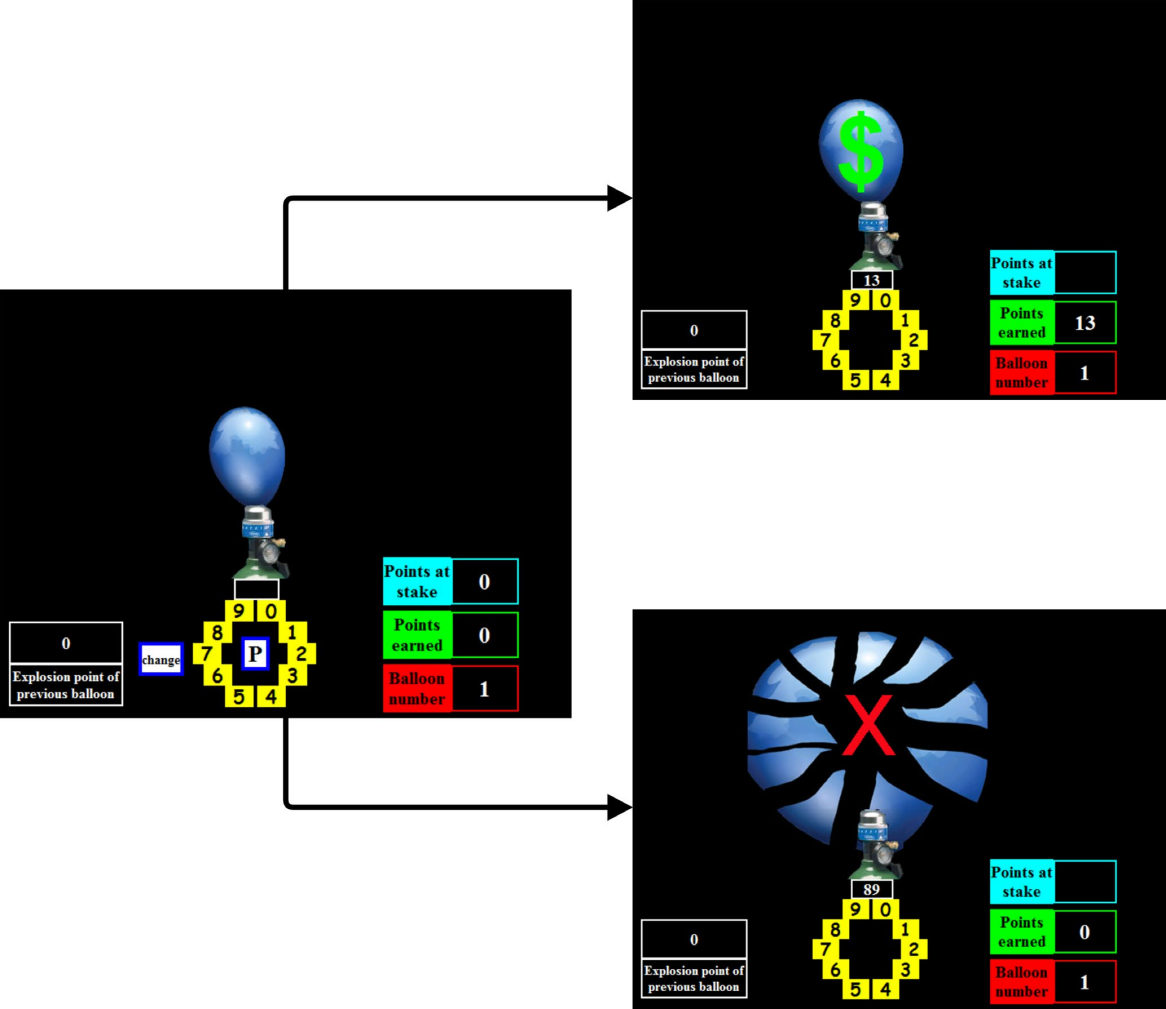
Task setup of the Balloon Analogue Risk Task (BART). The left screen shows the starting position. The upper right screen shows a situation in which the participant receives positive feedback. The lower right screen shows a situation where negative feedback is provided

In the automatic BART (Euser, Evans, et al., 2013; Euser, Greaves‐Lord, et al., 2013; Pleskac, Wallsten, Wang, & Lejuez, 2008; Yau et al., 2015), participants pump up a virtual balloon. More pumps equal more points but also increase the chance that the balloon pops, in which case all points accumulated in that trial are lost. The task setup is presented in Figure 1. On the left side of the screen, the explosion point of the previous balloon is shown. On the right, three parameters are provided: how many points are at stake in the present trial (blue box), how many points have been accumulated so far (green box), and the trial number (red box). In the middle, a number dial is shown. Participants had to pump up 60 balloons. Via the number dial, participants first indicated how many times they wanted to pump the balloon, ranging from 1 to 128. Then, they pressed “P”, after which the balloon started inflating. After inflation, either positive or negative feedback followed. In case of positive feedback, the balloon remained intact and a green dollar sign appeared (Figure 1, upper right). In case of negative feedback, the balloon popped and a red cross appeared (Figure 1, lower right). The explosion likelihood distribution was equal for every possible pump, with an average of 64 pumps, and was the same for all participants. As in the original BART, participants were not informed about the explosion likelihood distribution. The variable of interest for the BART was the average number of pumps across all trials.

#### 2.4.2 Cold and hot Columbia Card Task (CCT)

**Figure 2:**
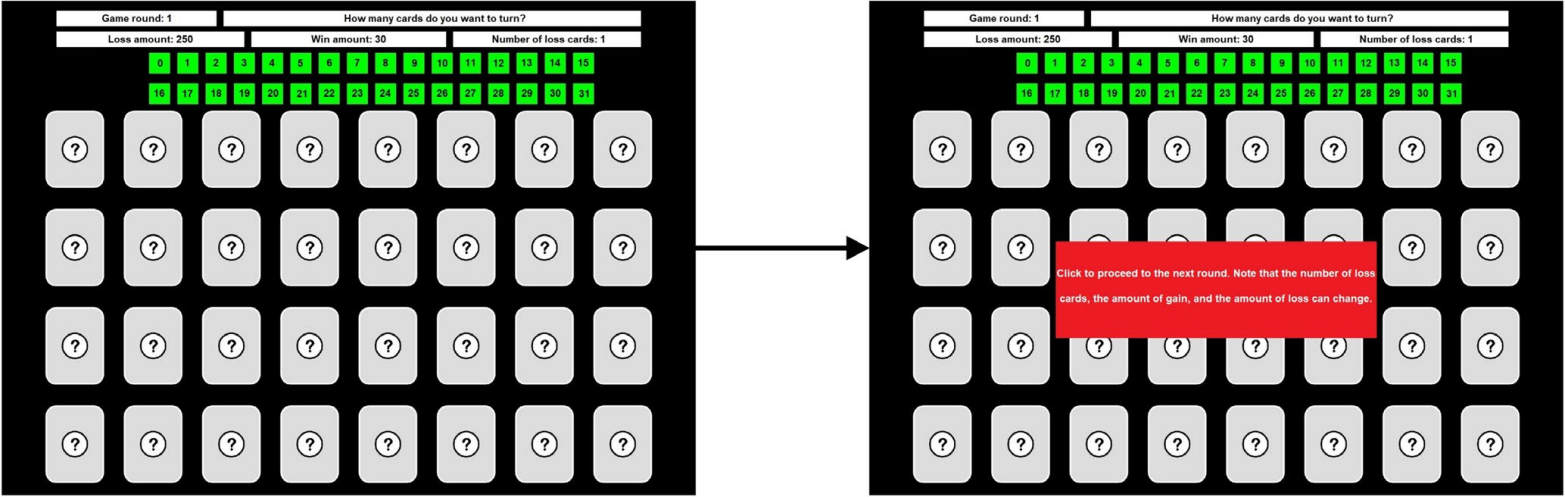
Task setup of the cold Columbia Card Task (CCT). The left screen represents the initial setup in which the participant indicates how many cards he/she wants to turn by clicking a number from 0 to 31. The right screen shows the message participants see after selecting a number, which informs them that the next round is about to start and that the information parameters may change

**Figure 3:**
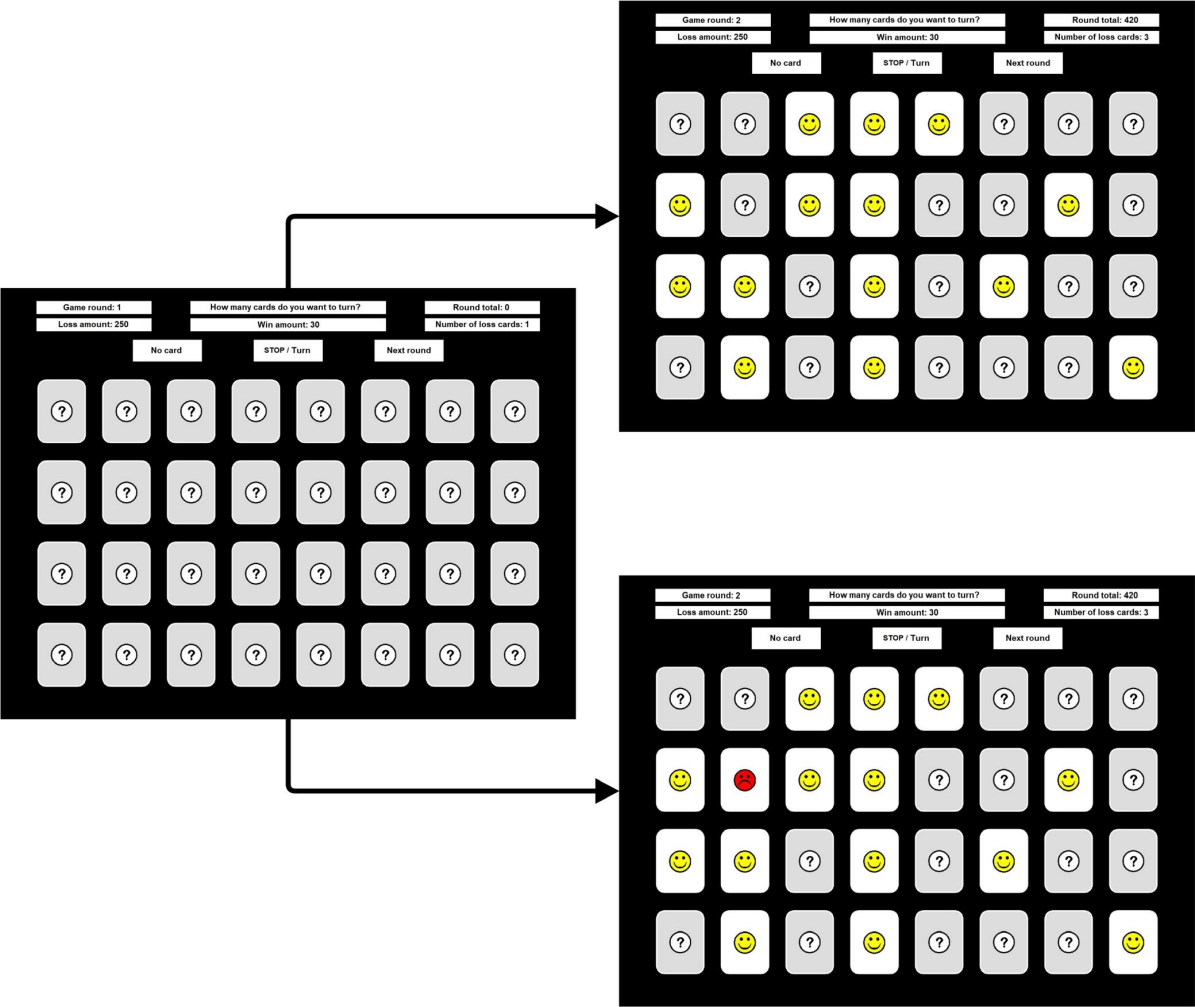
Task setup of the hot Columbia Card Task (CCT). The left screen represents the initial setup the participant encounters. The upper right screen shows the setup during turning the cards. The lower right screen shows the setup when a loss card is encountered

In the cold and hot CCT (Figner et al., 2009), participants are presented with a virtual array of 32 (4 × 8) cards. The majority of these cards earn the participant points (win cards), but in every array a small number of loss cards is hidden, for which points are subtracted. In the cold CCT, participants choose the total number of cards they would like to turn at the start of every round by clicking a number from 0 to 31. After selecting a number, a message appears, informing participants that they are continuing to the next round. This task setup is presented in Figure 2. No feedback is provided during the cold CCT; participants are only informed about their final points after finishing the task. In the hot CCT, in contrast, participants turn over cards one by one in a self‐paced manner and receive immediate feedback (i.e., without delay) in the form of a happy face (win card) or a sad face (loss card). Participants can decide to stop turning cards at any point, terminating the round. However, if they encounter a loss card (which is shown for 2,000 ms), the round terminates automatically, and the specified loss amount is subtracted from the points earned in that round. The task setup of the hot CCT is presented in Figure 3. In both the cold and the hot CCT, participants are given three information parameters to help them decide how many cards to turn: (1) the number of points they gain when turning a win card, (2) the number of points they lose when turning a loss card, and (3) the number of loss cards hidden in a round. These parameters are presented at the top of the screen and are independently varied across trials by means of a full factorial design. The number of levels within a parameter differs across studies, with most studies using two levels per parameter: 250 or 750 loss, 10 or 30 win, and 1 or 3 chance (Brunell & Buelow, 2017; Buelow, 2015; Holper & Murphy, 2014; Huang et al., 2013; Panno et al., 2013; Penolazzi et al., 2012; Pripfl et al., 2013; Schumpe et al., 2017). In the present study, we ran this 2 × 2 × 2 factorial six times, resulting in 48 trials $\left(\left[2\times 2\times 2\right]\times 6\right)$. Given that the focus of the present study is on the hot CCT, the only variable of interest extracted from the cold CCT was the absolute risk level (i.e., the number of cards chosen). The variables of interest for the hot CCT were the average number of card turns; the number of loss card encounters; and sensitivity to gains, losses, and probabilities. Since data from the hot CCT are inherently censored (i.e., people's observed risk level in trials that randomly forcedly end when turning a loss card does not necessarily reflect their true risk level), the variables of interest were in addition calculated using only data from trials in which participants voluntarily stopped turning cards.

### 2.5 Electrophysiological recordings and signal processing

EEG was recorded using a 32‐channel amplifier and ActiveTwo data acquisition software (Biosemi, Amsterdam, the Netherlands). Ag/AgCl active electrodes were placed on the scalp by means of a head cap according to the 10–20 placing system. The electrooculogram (EOG) was recorded by placing flat electrodes above and below the left eye (vertical EOG) and at the outer canthi of both eyes (horizontal EOG). Two reference electrodes were placed on the mastoids. An active (common mode sense) and passive (driven right leg) electrode comprised a feedback loop for amplifier referencing. All signals were digitized with a sampling rate of 512 Hz.

The data were analyzed offline using Brain Vision Analyzer 2 (Brain Products, Gilching, Germany). First, all EEG channels were referenced to the mathematically linked mastoid electrodes. Then we applied a high‐pass filter of 0.10 Hz, a low‐pass filter of 30.00 Hz, and a notch filter of 50.00 Hz (to filter out powerline artifacts). Data were segmented into epochs ranging from 100 ms before onset of the feedback presentation to 1,000 ms after onset of the feedback presentation. Then ocular artifact correction (Gratton, Coles, & Donchin, 1983) and baseline correction (using the 100 ms pre‐feedback presentation window) were applied. Finally, extreme amplitudes (below –75 µV or above 75 µV) were removed using automatic artifact rejection. The average number of segments used for calculating individuals’ total ERP was *M* = 172.80 following positive feedback (a happy icon) and *M* = 8.52 following negative feedback (a sad icon). The time window used for analyzing the FRN was 220–300 ms; the P300 was analyzed across a time window of 300–450 ms. All epochs were averaged across midline (Fz, Cz, Pz, Oz) and adjacent (F3, F4, C3, C4, P3, P4, O1, O2) electrodes in order to minimize myogenic artifacts.

### 2.6 Analyses

The first aim of the study was examining whether feedback appraisal after risky decision‐making in the CCT was accompanied by an FRN and P300, and whether these ERPs differed between positive and negative feedback. To this end, the averaged absolute and difference waves as recorded from 100 ms before to 1,000 ms after feedback presentation were plotted. In addition, two repeated‐measures analyses of variance (ANOVAs) (2 [valence: positive, negative] × 4 [cluster: frontal, central, parietal, occipital]) were performed to examine whether the ERP in response to positive feedback significantly differed from the ERP in response to negative feedback for the 220–300 FRN time period and for the 300–450 P300 time period. A Bonferroni‐corrected 5% alpha level was used. Since repeated‐measures ANOVAs are susceptible to violation of the sphericity assumption, this assumption was tested using Mauchly's test of sphericity. Preempting the findings, the observed violations were relatively severe ($\hat{\varepsilon }\;<\;0.75$). Therefore, following the advice of Field (2013), these violations were corrected for by adjusting the degrees of freedom using the Greenhouse‐Geisser estimate.

The second research aim was to examine the validity of the CCT by pairwise correlating the EEG difference waves with risk‐related self‐reports and behavioral constructs: gender; average number of hot CCT card turns; number of hot CCT loss card encounters; sensitivity to gain, loss, and probability information (calculated using per‐trial correlations between gain/loss/probability values and the number of cards chosen); average number of cold CCT cards; average number of BART balloon pumps; and self‐reported reward responsiveness, impulsiveness, and sensation‐seeking. Given the relatively large number of correlations (22), we would expect one correlation to be wrongly marked as “significant”. Because of the limitations associated with significance testing (Cohen, 1990), we also focused on the magnitude of the effects (the correlation coefficients).

Finally, three sets of robustness analyses were performed. The first robustness check examined whether the correlational findings lasted when the behavioral CCT data were solely based on trials in which participants voluntarily stopped turning cards (hence trials for which data was uncensored). A second check examined the robustness of the ERPs itself and their correlations with the self‐reports and behavioral constructs when excluding (1) individuals who reported a current psychiatric or neurological disorder, (2) individuals whose FRN and/or P300 difference scores were “reversed” (i.e., the opposite of what was expected, namely a stronger response to positive than negative feedback), and (3) individuals with univariate and/or bivariate outlying values as detected via visual inspection of the histograms, boxplots, and scatterplots, and by checking for extreme (> |3.29|) standardized residuals. In a third check, we abandoned the difference wave approach and examined correlations between self‐report/behavioral measures and absolute ERPs (i.e., the separate measures for FRN gain, FRN loss, P300 gain, and P300 loss). Given that a difference score is computed using absolute scores, its correlation may be conflated, and its interpretation may be unclear (Meyer, Lerner, de los Reyes, Laird, & Hajcak, 2017). In particular, if the gain and loss ERPs correlate with each other but are correlated with risk‐taking in opposite directions, individual correlations are suppressed when using difference scores. This last robustness check examined whether this was the case for the present data.

## 3 RESULTS

### 3.1 Visual representation and interpretation of the ERPs

**Figure 4:**
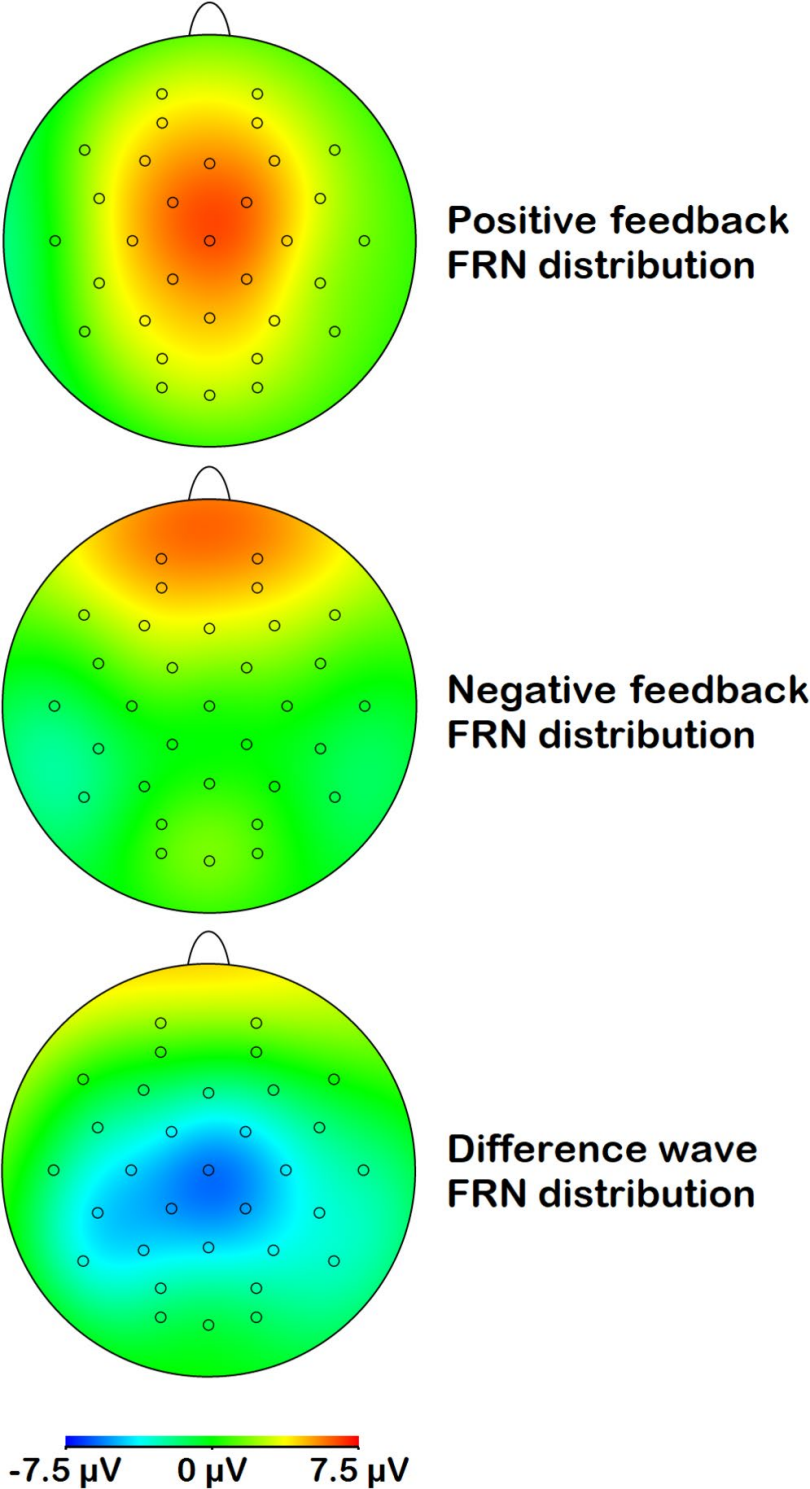
Topographical distribution of FRN activity across the scalp for positive feedback, negative feedback, and the difference between these two (negative minus positive)

**Figure 5:**
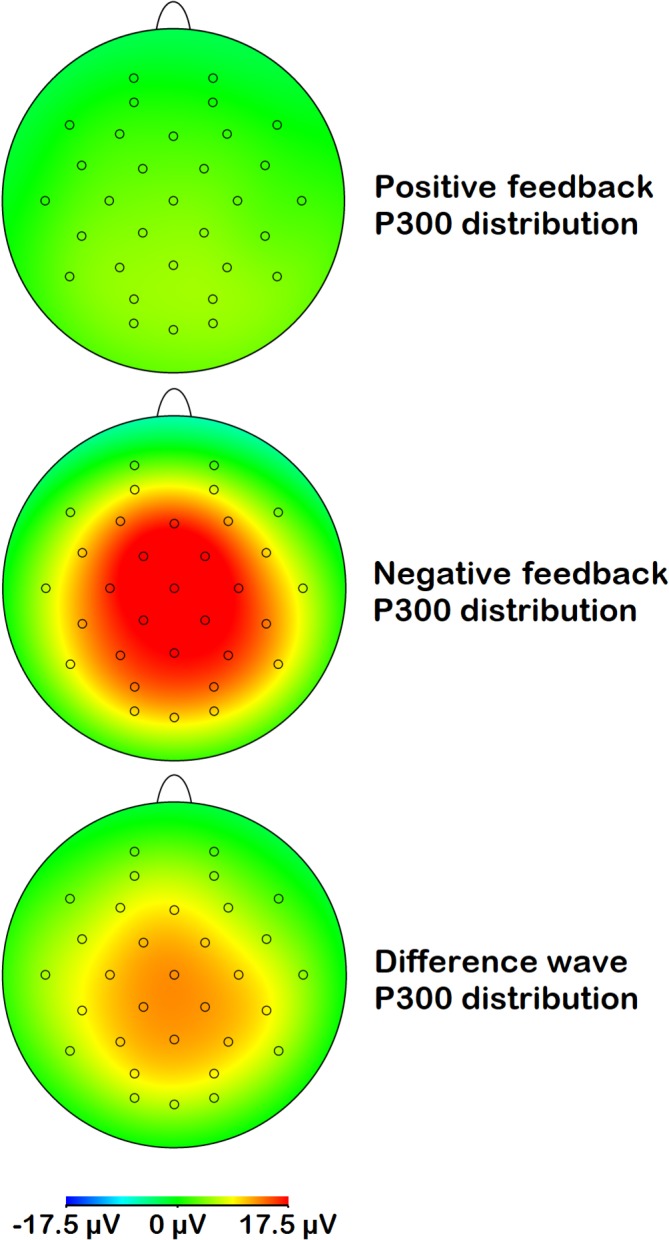
Topographical distribution of P300 activity across the scalp for positive feedback, negative feedback, and the difference between these two (negative minus positive)

Figures 4 and 5 show the scalp distributions of respectively the FRN and the P300 for positive feedback, negative feedback, and the difference wave. FRN activity peaked at frontocentral sites, while its difference activity was located more parietally. P300 activity showed a central‐parietal distribution.

**Figure 6:**
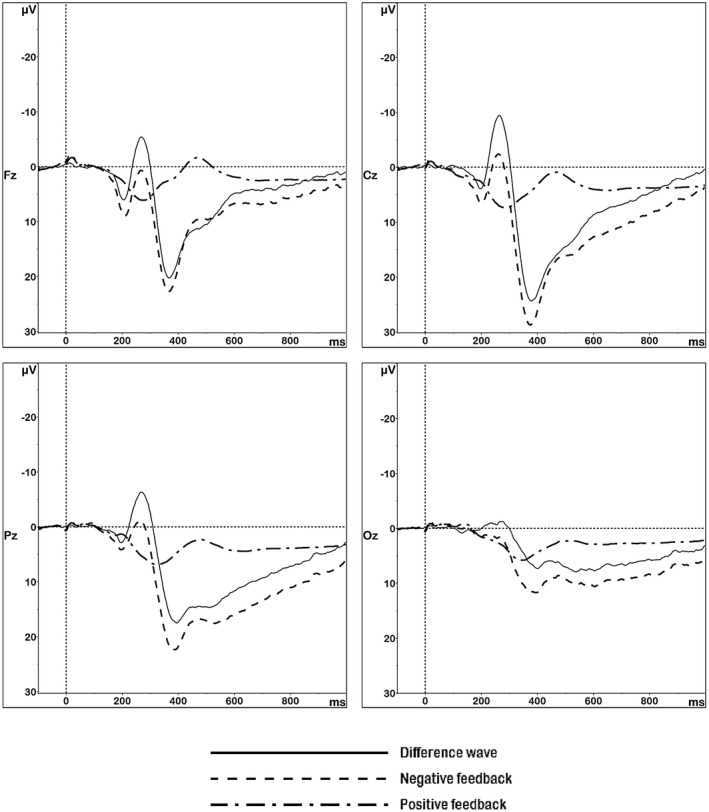
Grand averaged ERPs for the hot Columbia Card Task (CCT). The dash‐dotted line represents the electrophysiological response to positive feedback; the dashed line represents the electrophysiological response to negative feedback; and the solid line is the calculated difference wave

The grand averaged ERP waveforms are presented in Figure 6. The waveform appeared robust, with a clear FRN in the 220–300 ms window and a clear P300 in the 300–450 ms window, both of which were stronger following negative than following positive feedback. These observations were confirmed by the repeated‐measures ANOVAs, which were (after discarding ERP segments in the preprocessing phase) based on data from *n* = 121 individuals. For the FRN, the Greenhouse‐Geisser correction was applied to the main effect of cluster (χ^2^(5) = 269.65, *p* < 0.001, $\hat{\varepsilon }\;=\;0.50$) and to the interaction (χ^2^(5) = 214.20, *p* < 0.001, $\hat{\varepsilon }\;=\;0.56$). The main effects showed that the potential was stronger in response to negative than to positive feedback (∆2.70 µV, *F*(1, 120) = 31.72, *p* < 0.001, η^2^_p_ = 0.21), and that it differed across clusters (*F*(1.50, 179.96) = 25.55, *p* < 0.001, η^2^_p_ = 0.18). These factors interacted as well (*F*(1.68, 201.97) = 28.24, *p* < 0.001, η^2^_p_ = 0.19): the effect of valence was strongest at central (∆4.53 µV) and parietal (∆3.77 µV) electrodes, and weaker at frontal (∆1.24 µV) and occipital (∆1.25 µV) ones. For the P300, the Greenhouse‐Geisser correction was again applied to the main effect of cluster (χ^2^(5) = 215.28, *p* < 0.001, $\hat{\varepsilon }\;=\;0.62$) and to the interaction (χ^2^(5) = 204.98, *p* < 0.001, $\hat{\varepsilon }\;=\;0.60$). The main effects confirmed that the potential was stronger in response to negative than to positive feedback (∆10.57 µV, *F*(1, 120) = 352.87, *p* < 0.001, η^2^_p_ = 0.75), and that it differed across clusters (*F*(1.87, 223.94) = 85.60, *p* < 0.001, η^2^_p_ = 0.42). The interaction (*F*(1.80, 215.69) = 126.95, *p* < 0.001, η^2^_p_ = 0.51) showed that the difference in valence was weaker at occipital (∆4.90 µV) than at central (∆14.57 µV), frontal (∆11.72 µV), and parietal (∆11.09 µV) sites.

### 3.2 Correlational analyses

Table 1 shows the correlations between the FRN and P300 difference waves on the one hand and the risk‐related self‐reports and behavioral constructs on the other. The correlations involving gender are point‐biserial (*r_pb_*); the remaining correlations are bivariate (*r*). Analyses were performed pairwise on an *n* between 119 and 121, with most missing values resulting from incomplete surveys or missing ERP data. None of the correlations was significant at a 5% level, which was fewer than the one significant correlation that was expected by chance. The majority of the effects were small but had the expected direction.

**Table 1:** Correlations between the FRN and P300 difference waves and risk‐related self‐reports and behavioral constructs

	*M*	*SD*	Min	Max	Correlations	
					FRN CCT difference wave	P300 CCT difference wave
Gender (male = 0)	0.52	0.50	0.00	1.00	0.09^a^	–0.04^a^
Hot CCT average card turns	7.25	2.18	1.65	12.65	0.03	–0.01
Hot CCT loss card encounters	22.21	8.31	6.00	44.00	0.05	–0.06
Hot CCT gain sensitivity	0.16	0.18	–0.22	0.57	–0.10	0.16
Hot CCT loss sensitivity	–0.22	0.18	–0.64	0.22	0.14	–0.05
Hot CCT probability sensitivity	–0.51	0.12	–0.86	–0.11	0.01	–0.05
Cold CCT number of chosen cards	8.57	3.71	1.60	21.81	0.10	0.07
BART number of chosen pumps	58.49	11.72	13.42	85.25	0.09	0.13
Reward responsiveness	16.63	2.15	9.00	20.00	–0.08	–0.16
Impulsiveness	65.46	8.32	43.00	87.00	0.13	–0.03
Sensation seeking	25.48	5.62	11.00	37.00	0.09	–0.04

a Point‐biserial correlations (*r_pb_*).

Since the FRN is a negative potential, its difference score is generally negative. Therefore, we expected a positive correlation with risk‐taking (i.e., the more negative the FRN difference score, the smaller self‐reported or behavioral risk‐taking, and vice versa). In line with this, smaller FRN difference scores (reflecting reduced electrophysiological response to the feedback) were correlated with encountering more loss cards in the hot CCT (*r* = 0.05), choosing more cards in the cold (*r* = 0.10) and hot (*r* = 0.03) CCT, pressing more balloon pumps in the BART (*r* = 0.09), being less sensitive to information on losses (*r* = 0.14), and reporting higher impulsiveness (*r* = 0.13) and sensation‐seeking (*r* = 0.09). Three correlations were in the opposite direction of what was expected, namely those for gender (*r* = 0.09), sensitivity to gains (*r* = –0.10), and reward responsiveness (*r* = –0.08).

Since the P300 is a positive potential, its difference scores are generally positive, and hence negative correlations were expected between this difference score and risk‐taking (i.e., the more positive the P300 difference score, the smaller self‐reported or behavioral risk‐taking, and vice versa). In line with this, smaller P300 difference scores (reflecting reduced electrophysiological response to the feedback) were correlated with encountering more loss cards in the hot CCT (*r* = –0.06), being less sensitive to information on losses (*r* = –0.05) and probabilities (*r* = –0.05), and reporting higher reward responsiveness (*r* = –0.16), impulsiveness (*r* = –0.03), and sensation seeking (*r* = –0.04). Four correlations were in opposite direction of what was expected, namely those for gender (*r* = –0.04), sensitivity to gains (*r* = 0.16), cold CCT cards chosen (*r* = 0.07), and BART balloon pumps (*r* = 0.13).

### 3.3 Robustness checks

The first robustness analysis examined whether the correlational findings lasted when only using uncensored behavioral CCT data. To this end, eight correlations were rerun, namely those including the average number of card turns in the hot CCT, gain sensitivity, loss sensitivity, and probability sensitivity. Half of these correlations (most relatively large) remained similar in size and direction; three (all of them small) changed direction; and one changed substantially in size, namely the correlation between hot CCT card turns and the P300 difference wave, which changed from –0.01 to –0.10 and thereby became more in line with the hypothesis that a smaller P300 difference wave is associated with taking more risk (see Table S1).

A second set of robustness checks examined the effect of participant exclusion (Tables S2–S4, Figures S1–S3). The exclusion of individuals with psychiatric or neurological disorders (*n* = 5) did not impact the direction of the correlations, nor did it substantially change correlation size. The same was true for exclusion of individuals with outlying values (*n* = 5), except for the correlation of the P300 difference wave with impulsiveness (which changed direction: from *r* = –0.03 to *r* = 0.05) and with the BART (which turned significant: from *r* = 0.13 to *r* = 0.20). Neither exclusion impacted the grand averaged waveform or the topographical distribution of the ERPs. The largest change in findings was observed when excluding individuals with reversed difference scores (*n* = 48). This caused a moderate increase in FRN amplitude and in most FRN difference score correlations, which became more in line with our hypotheses, such as the correlation with hot CCT card turns (from *r* = 0.03 to *r* = 0.20), loss card encounters (from *r* = 0.05 to *r* = 0.20), and loss sensitivity (from *r* = 0.14 to *r* = 0.30). The correlations for the P300 difference wave, however, changed in a less consistent manner and overall became less in line with our hypotheses. Since most exclusions resulted from reversed FRN scores, the impact on P300 correlations may indeed have been more equivocal (by also discarding “regular” scores).

The third and final set of robustness checks examined whether the use of difference scores suppressed the correlations between the absolute ERPs and the risk‐related self‐reports and behavioral constructs. The gain and loss FRN ERPs were correlated with each other (*r* = 0.31), as were the gain and loss P300 ERPs (*r* = 0.37). However, few of the gain and loss ERPs correlated with risk‐related constructs in opposite direction (5 out of 11 for the FRN and 3 out of 11 for the P300), hence indicating no major risk of conflated difference scores. Examination of the individual correlations (Table S5) showed that the majority of FRN loss and P300 gain correlations were not in line with expectations, and hence did not outperform the correlations based on difference scores. However, most FRN gain and P300 loss correlations were in line with expectations. A stronger FRN in response to gains was associated with being male (*r* = 0.19), higher self‐reported and behavioral risk‐taking (*r* = –0.02 to *r* = –0.23), and lower focus on loss (*r* = –0.19) and probability (*r* = –0.10) information. A stronger P300 in response to losses was associated with being female (*r* = 0.05), lower self‐reported and behavioral risk‐taking (*r* = –0.06 to *r* = –0.13), and a stronger focus on information on loss (*r* = –0.10) and probability (*r* = –0.07). Thus, the FRN gain and P300 loss correlations provided information beyond that offered by the correlations for the difference scores.

## 4 DISCUSSION

The present study examined ERPs in response to feedback in the hot version of the CCT (Figner et al., 2009). In line with research on feedback‐related ERPs in for example the IGT and the BART, feedback appraisal in the CCT was accompanied by a clear FRN and P300, which were stronger in response to losses than to gains. This pattern did not change after excluding individuals with psychiatric or neurological disorders, individuals with outlying scores, or individuals with reversed (positive > negative) ERP difference scores. Hence the ERPs appeared robust. Despite this, correlations between the ERP difference waves and risk‐related self‐reports and behavioral measures were nonsignificant and small. Most correlations did show an effect in the expected direction though: for example, smaller FRN difference scores were associated with taking more risk in the cold CCT, decreased sensitivity to information on losses, and higher impulsiveness; smaller P300 difference scores were most strongly associated with higher reward responsiveness. When correlating absolute instead of difference scores, the FRN gain and P300 loss (but not the FRN loss and P300 gain) also showed effects in the expected direction. Excluding individuals with reversed ERP difference waves strengthened most FRN correlations, thereby bringing the findings more in line with the hypotheses. Several possible explanations exist why individuals show such a reversed pattern. First, they may respond very weakly to losses and/or very strongly to gains, resulting in a more potent ERP in response to gains than to losses. Alternatively, these reversed waves may result from individuals’ expectations. Especially the FRN has been suggested to represent a reward prediction error, an indicator of the difference between expected and observed outcomes (Holroyd & Coles, 2002; Sambrook & Goslin, 2015). Hence, encountering losses may elicit only modest ERPs when they are expected, compared to cases in which they are not. Given the impact participants with reversed scores had on the correlations in the robustness analyses, future studies may want to address this phenomenon and examine its underlying causes.

This study's results combined with previous findings also illustrate some challenges that are more specific to the CCT and that would benefit from further investigation. First, for studies using the CCT in combination with EEG, it will be key to have a more elaborate understanding of which (task) characteristics influence the ERPs, and whether such influence is desirable. In addition to the large sample size, one factor possibly influencing the strength and robustness of the CCT's potentials concerns the reward structure. Risk tasks can offer participants different types of incentive, such as monetary rewards (Xu et al., 2016), social rewards (Op de Macks et al., 2017), or sexual rewards (Lawyer, 2013). Moreover, tasks differ in how participants are penalized when losing a trial: it may result in not receiving any reward, or in losing a reward that was acquired before. In the CCT, the points participants earn are truly at stake since turning a loss card means that the specified loss amount is subtracted from the points earned. Furthermore, participants in the present study were offered real (vs. hypothetical) money, which increased the ecological validity of the task and which has been shown to elicit a stronger FRN for negative feedback (Xu et al., 2016). This combination of losing points that represent real money may induce a larger prediction error and therefore stronger ERPs. This contribution to the presently observed robust ERPs can be deemed desirable as it reflects the constructs presumed to underlie these ERPs. A second factor that may have influenced the ERPs seems less desirable: the stimulus‐sequence history. In the CCT, most loss‐card encounters are preceded by a series of win card encounters, with losses being roughly 20 times less frequent than wins. Such oddball structures have been shown to impact ERPs: FRN amplitudes tend to be larger when successive encounters with a stimulus are followed by feedback of opposite valence compared to feedback of the same valence (Holroyd & Coles, 2002), and P300 amplitudes are larger after presenting a deviant or salient stimulus, especially when this stimulus is preceded by a series of other stimuli (Nieuwenhuis et al., 2005; Squires, Wickens, Squires, & Donchin, 1976). The oddball‐like structure of the CCT can reasonably have contributed to the strength of the ERPs (especially the negative‐feedback waveform), thereby adding unintended systematic variance that future studies may want to mitigate.

In addition to this EEG‐related concern, the hot version of the CCT poses a more general challenge: censoring. As briefly discussed in the method section, data from the hot CCT are inherently censored as people's observed risk level in trials in which they turn a loss card does not necessarily reflect their true risk level, since they might have taken more risk (i.e., turned more cards) if they had had the chance to do so. In the present study, we accommodated for censoring by running two sets of analyses: the main analyses, using data from all trials; and robustness analyses, using only data from trials in which participants had voluntarily stopped turning cards. A similar approach was employed by Kluwe‐Schiavon et al. (2015), who reported no major changes in their final results. In the present study, one of eight rerun correlations substantially changed in size, demonstrating the effect that censoring can have on a study's findings. An alternative solution to censoring is offered by Figner et al. (2009), who prevented censoring ex ante by rigging the task. In their task setup, 54 experimental trials are supplemented by nine trick trials. In the experimental trials, the loss card is programmed to be the last possible card, so that participants never encounter it and so that all stopping points are voluntary. These uncensored data are used for analysis. Credibility is upheld by randomly interspersing the nine trick trials, which are programmed in such a way that participants quickly encounter a loss card. However, Figner et al.’s (2009) solution to censoring seems problematic, as the rigged percentage of trials in which participants encounter a loss card seems unrealistically low: $9÷\left[54+9\right])\times 100\approx 14.29$. In the present unrigged study, participants on average encountered a loss card in 22.21÷48×100≈46.27 percent of trials, which was shown to be significantly higher using a one sample *t*‐test: *t*(125) = 20.73, *p* < 0.001. Arguably, presenting people with (too) little negative feedback could cause them to take more risk. Tentative evidence for this conjecture is found in the large difference between risk levels found in studies using a rigged CCT (~23 cards [Figner et al., 2009], 27 cards [Markiewicz & Kubińska, 2015], and 21 cards [Penolazzi et al., 2012]) and studies in which the cards are truly shuffled (7.25 [8.48 uncensored] in the present study, and ~12 in Holper & Murphy, 2014).

A final, easier solution to censoring is omitting the hot CCT altogether and instead using the warm CCT, which also measures affective risk‐taking but delays the feedback, so that participants first decide how many cards they want to turn and only then observe the (per‐card) outcome of their decision. Although this does solve the issue of censoring, it offers no solution to the other challenge we discussed with regard to the CCT, that is, its oddball structure. Notably, the rigged design by Figner et al. (2009) does solve the oddball problem (at least within the trial). Whereas loss card encounters in an unrigged design are generally preceded by a series of win cards, loss card encounters in Figner et al.’s (2009) design are artificially positioned at the start of trials, thereby mitigating the oddball effect. This does not change the fact that win cards are more frequent than loss cards across the task but does impact the local probability. Therefore, if the percentage of rigged trials in which participants encounter a loss card in Figner et al.’s (2009) design were set to a more plausible, naturalistic value, both challenges observed in the present study would be resolved: the influence of the hot CCT's oddball structure on its ERPs and its masking of true values in trials that forcedly end (censoring). We recommend future studies to keep these two challenges and our proposed solutions to them in mind when further validating or using the CCT either in a behavioral study or in combination with a (neuro)biological measure such as EEG.

## CONFLICT OF INTEREST

The authors declare to have no conflict of interest.
