# Supplementary material for: Event‐related potentials in response to feedback following risk‐taking in the hot version of the Columbia Card Task
Source: Psychophysiology. 2019 May 8;56(9):e13390. doi: 10.1111/psyp.13390 (PMC6850144; doi:10.1111/psyp.13390)
Supplement: Supplementary file 1 [file PSYP-56-na-s001.docx]

**Supplementary materials**

**Event-Related Potentials in Response to Feedback Following Risk-Taking in the Hot Version of the Columbia Card Task**

**Robustness 1 – Uncensored Behavioural CCT Data**

Table S1.

*Correlations Using Uncensored Behavioural CCT Data*

|  |  |  |  |  |  | Correlations | |
| --- | --- | --- | --- | --- | --- | --- | --- |
|  | *M* | *SD* | Min | Max |  | FRN CCT difference wave | P300 CCT difference wave |
| Hot CCT average card turns | 8.49 | 4.40 | 1.45 | 27.00 |  | -.03 | -.10 |
| Hot CCT gain sensitivity | 0.21 | 0.27 | -0.61 | 0.85 |  | -.11 | .13 |
| Hot CCT loss sensitivity | -0.31 | 0.24 | -0.81 | 0.54 |  | .17 | .04 |
| Hot CCT probability sensitivity | -0.58 | 0.18 | -0.94 | -0.07 |  | -.04 | -.06 |

Note: Green correlations indicate *r* ≥ |.03| correlations that were in the expected direction; orange correlations indicate *r* ≥ |.03| correlations that were in opposite direction.

**Robustness 2 – Exclusions**

Table S2.

*Correlations After Excluding Five Participants with a Current Psychiatric or Neurological Disorder*

|  |  |  |  |  |  | Correlations | |
| --- | --- | --- | --- | --- | --- | --- | --- |
|  | *M* | *SD* | Min | Max |  | FRN CCT difference wave | P300 CCT difference wave |
| Gender (male = 0) | 0.53 | 0.50 | 0.00 | 1.00 |  | .12^a^ | -.03^a^ |
| Hot CCT average card turns | 7.31 | 2.15 | 1.65 | 12.65 |  | .05 | -.02 |
| Hot CCT loss card encounters | 22.39 | 8.23 | 6.00 | 44.00 |  | .07 | -.06 |
| Hot CCT gain sensitivity | 0.16 | 0.17 | -0.22 | 0.57 |  | -.12 | .18 |
| Hot CCT loss sensitivity | -0.22 | 0.18 | -0.64 | 0.22 |  | .14 | -.07 |
| Hot CCT probability sensitivity | -0.52 | 0.13 | -0.86 | -0.11 |  | <\|.01\| | -.05 |
| Cold CCT number of chosen cards | 8.56 | 3.66 | 1.60 | 21.81 |  | .08 | .07 |
| BART number of chosen pumps | 58.52 | 11.89 | 13.42 | 85.25 |  | .09 | .14 |
| Reward responsiveness | 16.60 | 2.16 | 9.00 | 20.00 |  | -.10 | -.14 |
| Impulsiveness | 65.31 | 8.38 | 43.00 | 87.00 |  | .12 | -.04 |
| Sensation seeking | 25.42 | 5.64 | 11.00 | 37.00 |  | .07 | -.06 |

Note: ^a^ point-biserial correlations (*r_pb_*). Green correlations indicate *r* ≥ |.03| correlations that were in the expected direction; orange correlations indicate *r* ≥ |.03| correlations that were in opposite direction.

Table S3.

*Correlations After Excluding Five Participants with Outlying Scores*

|  |  |  |  |  |  | Correlations | |
| --- | --- | --- | --- | --- | --- | --- | --- |
|  | *M* | *SD* | Min | Max |  | FRN CCT difference wave | P300 CCT difference wave |
| Gender (male = 0) | 0.52 | 0.50 | 0.00 | 1.00 |  | .09^a^ | -.04^a^ |
| Hot CCT average card turns | 7.31 | 2.13 | 2.54 | 12.65 |  | .03 | -.06 |
| Hot CCT loss card encounters | 22.15 | 7.90 | 6.00 | 44.00 |  | .06 | -.07 |
| Hot CCT gain sensitivity | 0.16 | 0.18 | -0.22 | 0.57 |  | -.10 | .16 |
| Hot CCT loss sensitivity | -0.21 | 0.18 | -0.64 | 0.22 |  | .14 | -.08 |
| Hot CCT probability sensitivity | -0.52 | 0.12 | -0.86 | -0.19 |  | .04 | -.04 |
| Cold CCT number of chosen cards | 8.48 | 3.57 | 1.60 | 16.94 |  | .10 | .11 |
| BART number of chosen pumps | 58.98 | 10.93 | 25.37 | 85.25 |  | .12 | .20* |
| Reward responsiveness | 16.76 | 1.94 | 10.00 | 20.00 |  | -.06 | -.15 |
| Impulsiveness | 65.46 | 8.13 | 46.00 | 87.00 |  | .16 | .05 |
| Sensation seeking | 25.66 | 5.65 | 11.00 | 37.00 |  | .09 | -.06 |

Note: ^a^ point-biserial correlations (*r_pb_*). * is significant at a 5% level. Green correlations indicate *r* ≥ |.03| correlations that were in the expected direction; orange correlations indicate *r* ≥ |.03| correlations that were in opposite direction.

Table S4.

*Correlations After Excluding 48 Participants with Reversed FRN and/or P300 Difference Scores*

|  |  |  |  |  |  | Correlations | |
| --- | --- | --- | --- | --- | --- | --- | --- |
|  | *M* | *SD* | Min | Max |  | FRN CCT difference wave | P300 CCT difference wave |
| Gender (male = 0) | 0.47 | 0.50 | 0.00 | 1.00 |  | .12^a^ | .01^a^ |
| Hot CCT average card turns | 7.33 | 1.98 | 2.54 | 11.63 |  | .20 | .06 |
| Hot CCT loss card encounters | 22.42 | 8.00 | 6.00 | 44.00 |  | .20 | -.07 |
| Hot CCT gain sensitivity | 0.18 | 0.16 | -0.21 | 0.57 |  | -.14 | .17 |
| Hot CCT loss sensitivity | -0.21 | 0.18 | -0.61 | 0.14 |  | .30** | -.13 |
| Hot CCT probability sensitivity | -0.52 | 0.12 | -0.86 | -0.11 |  | -.02 | -.05 |
| Cold CCT number of chosen cards | 8.57 | 3.75 | 1.60 | 21.81 |  | .09 | .03 |
| BART number of chosen pumps | 57.63 | 11.24 | 13.42 | 81.95 |  | <\|.01\| | .27* |
| Reward responsiveness | 16.71 | 2.13 | 9.00 | 20.00 |  | .04 | .12 |
| Impulsiveness | 64.83 | 8.06 | 46.00 | 83.00 |  | .16 | .13 |
| Sensation seeking | 25.14 | 5.82 | 11.00 | 37.00 |  | .12 | .04 |

Note: ^a^ point-biserial correlations (*r_pb_*). * is significant at a 5% level; ** is significant at a 1% level. Green correlations indicate *r* ≥ |.03| correlations that were in the expected direction; orange correlations indicate *r* ≥ |.03| correlations that were in opposite direction.

| **Original** | **Psych/neuro** | **Outlier** | **Reversed** |
| --- | --- | --- | --- |
| 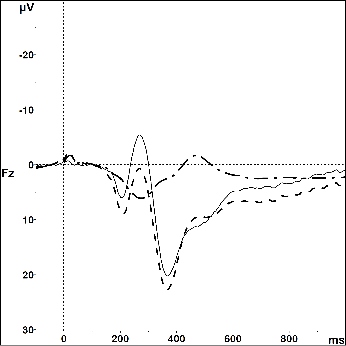 | 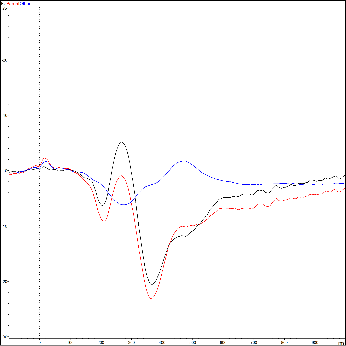 | 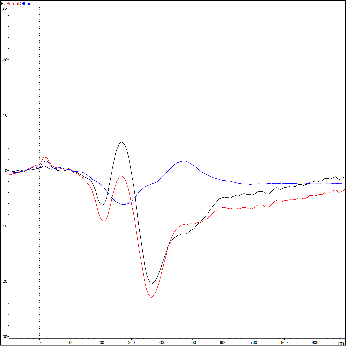 | 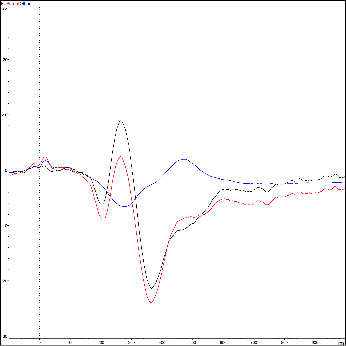 |
| 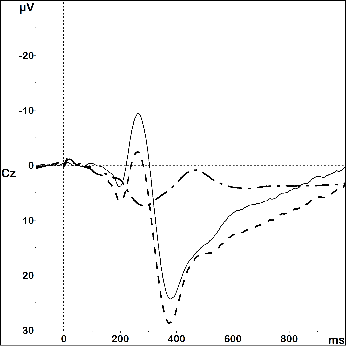 | 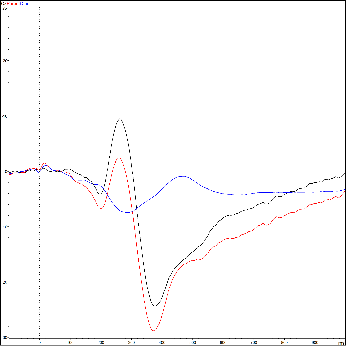 | 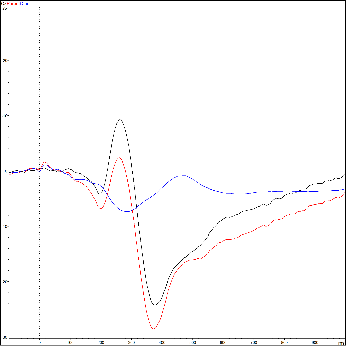 | 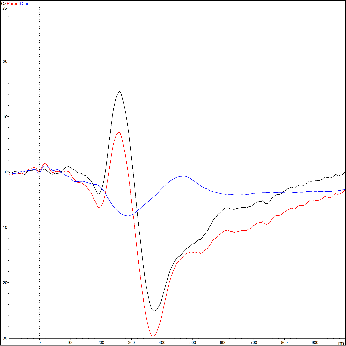 |
| 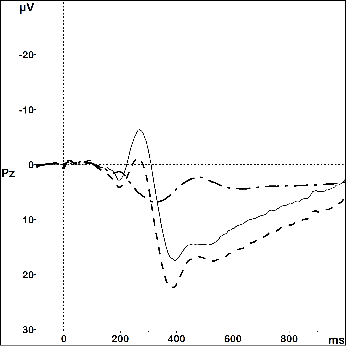 | 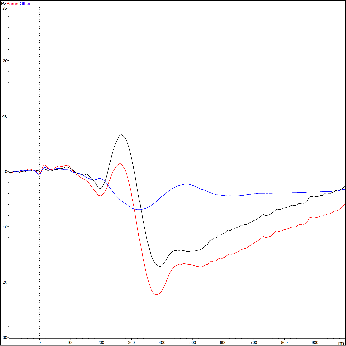 | 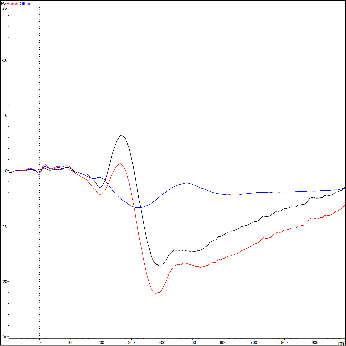 | 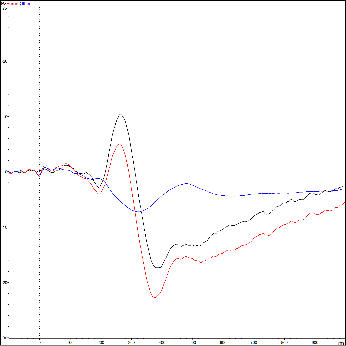 |
| 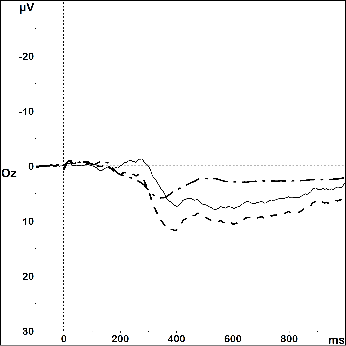 | 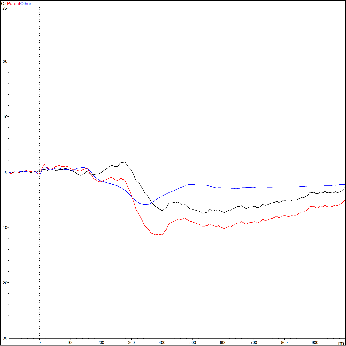 | 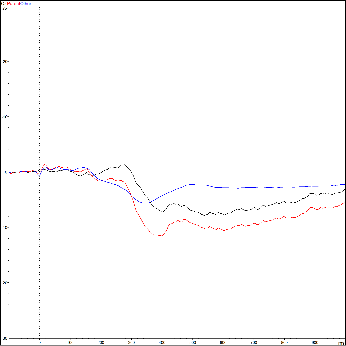 | 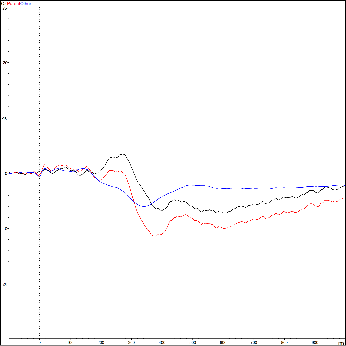 |

*Figure S1.* Effect of exclusions on the ERPs.

|  | **Original** | **Psych/neuro** | **Outlier** | **Reversed** |
| --- | --- | --- | --- | --- |
| **Pos** | 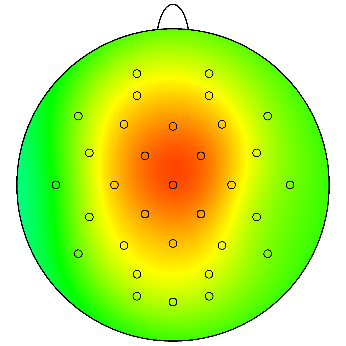 | 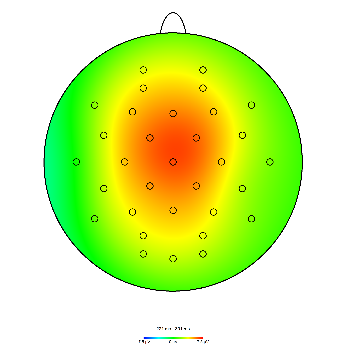 | 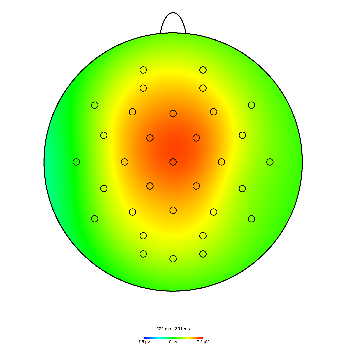 | 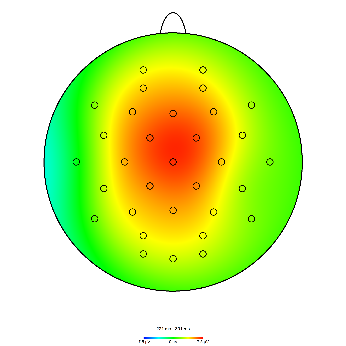 |
| **Neg** | 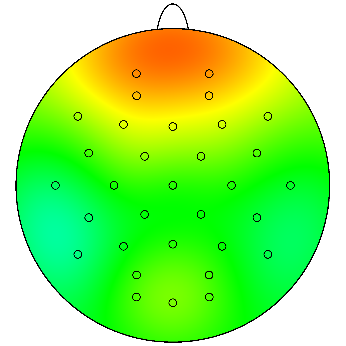 | 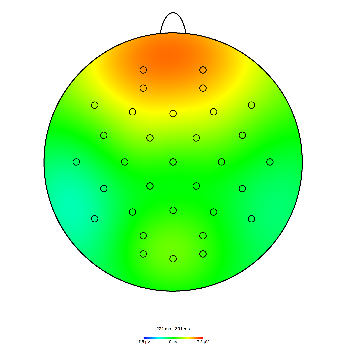 | 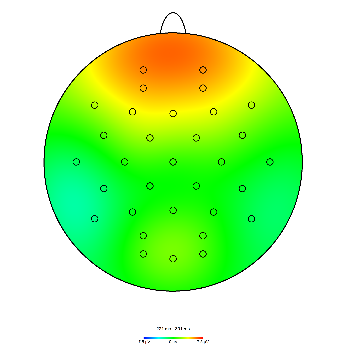 | 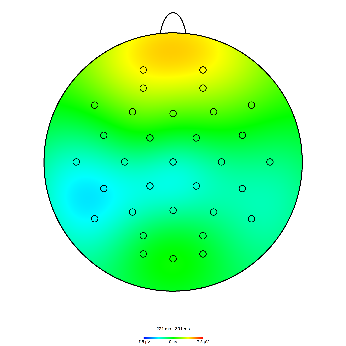 |
| **Diff** | 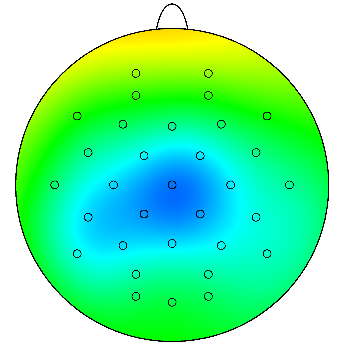 | 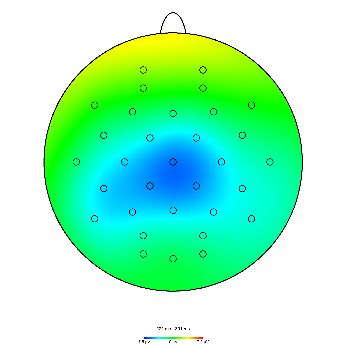 | 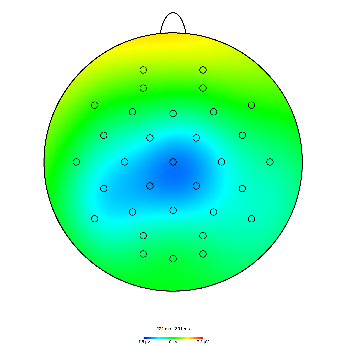 | 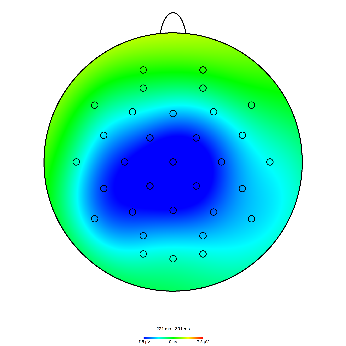 |

*Figure S2.* Effect of exclusions on the spatial distribution of the FRN.

|  | **Original** | **Psych/neuro** | **Outlier** | **Reversed** |
| --- | --- | --- | --- | --- |
| **Pos** | 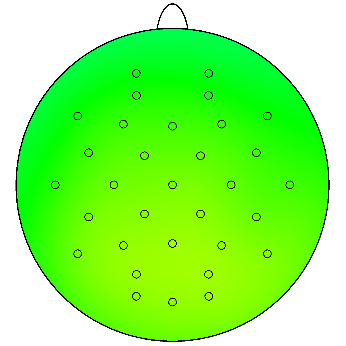 | 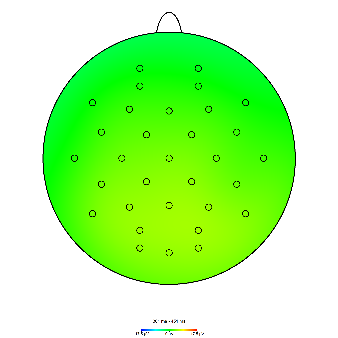 | 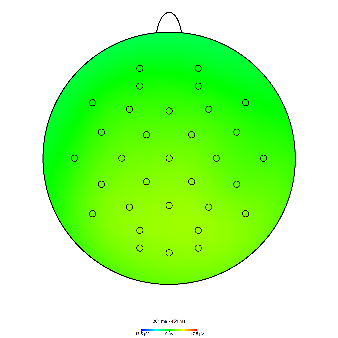 | 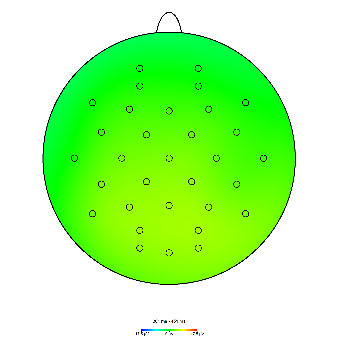 |
| **Neg** | 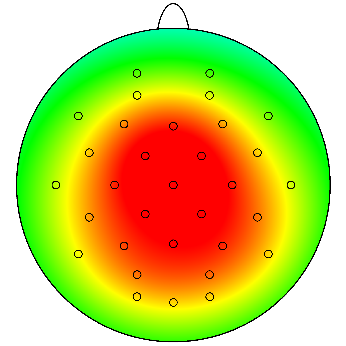 | 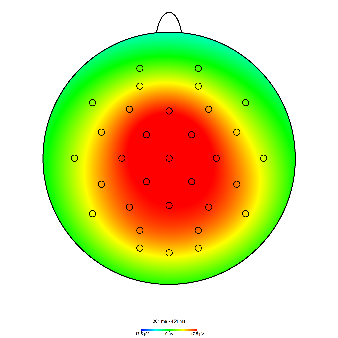 | 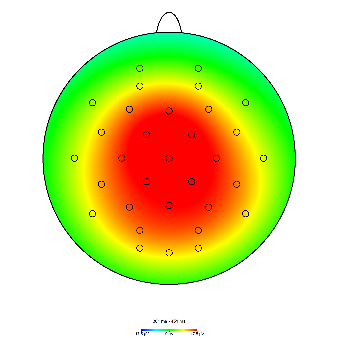 | 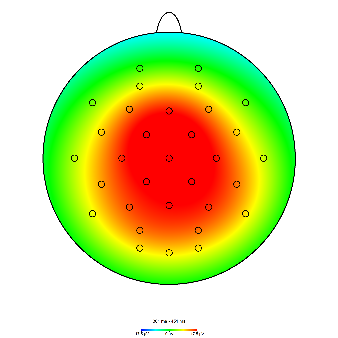 |
| **Diff** | 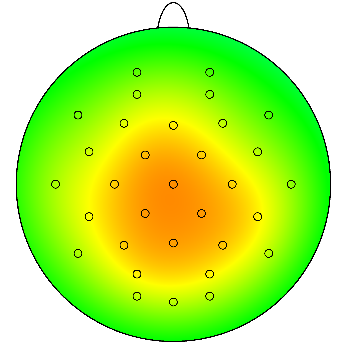 | 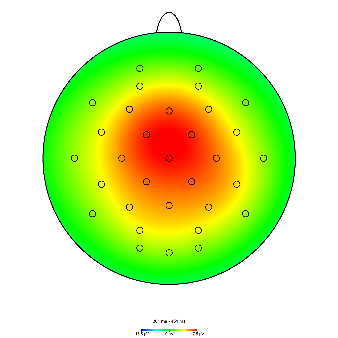 | 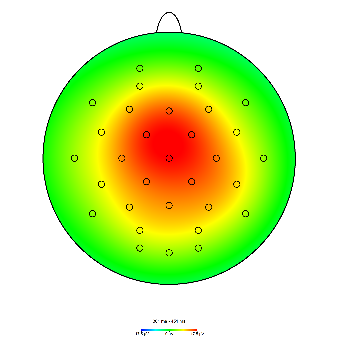 | 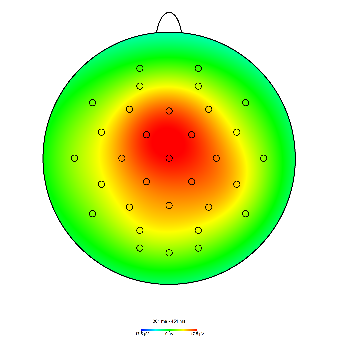 |

*Figure S3.* Effect of exclusions on the spatial distribution of the P300.

**Robustness 3 – Absolute ERPs**

Table S5. *Correlations Using Absolute ERPs*

|  |  |  |  |  |  | Correlations | | | |
| --- | --- | --- | --- | --- | --- | --- | --- | --- | --- |
|  | *M* | *SD* | Min | Max |  | FRN gain | FRN loss | P300 gain | P300 loss |
| Gender (male = 0) | 0.52 | 0.50 | 0.00 | 1.00 |  | .19^a^* | .17^a^ | .25^a^** | .05^a^ |
| Hot CCT average card turns | 7.25 | 2.18 | 1.65 | 12.65 |  | -.23** | -.09 | -.18* | -.09 |
| Hot CCT loss card encounters | 22.21 | 8.31 | 6.00 | 44.00 |  | -.22* | -.05 | -.14 | -.11 |
| Hot CCT gain sensitivity | 0.16 | 0.18 | -0.22 | 0.57 |  | .13 | -.01 | .10 | .21* |
| Hot CCT loss sensitivity | -0.22 | 0.18 | -0.64 | 0.22 |  | -.19* | .07 | -.14 | -.10 |
| Hot CCT probability sensitivity | -0.51 | 0.12 | -0.86 | -0.11 |  | -.10 | -.04 | -.08 | -.07 |
| Cold CCT number of chosen cards | 8.57 | 3.71 | 1.60 | 21.81 |  | -.05 | .08 | -.05 | .04 |
| BART number of chosen pumps | 58.49 | 11.72 | 13.42 | 85.25 |  | -.02 | .09 | -.06 | .10 |
| Reward responsiveness | 16.63 | 2.15 | 9.00 | 20.00 |  | -.06 | -.09 | .01 | -.13 |
| Impulsiveness | 65.46 | 8.32 | 43.00 | 87.00 |  | -.22* | .03 | -.25** | -.11 |
| Sensation seeking | 25.48 | 5.62 | 11.00 | 37.00 |  | -.14 | <\|.01\| | -.06 | -.06 |

Note: ^a^ point-biserial correlations (*r_pb_*). * is significant at a 5% level; ** is significant at a 1% level.
